# Modularity of PGL_2_(𝔽*p*)-representations over totally real fields

**DOI:** 10.1073/pnas.2108064118

**Published:** 2021-08-12

**Authors:** Patrick B. Allen, Chandrashekhar B. Khare, Jack A. Thorne

**Affiliations:** ^a^Department of Mathematics and Statistics, McGill University, Montreal, QC H3A 0B9, Canada;; ^b^Department of Mathematics, University of California, Los Angeles, CA 90095;; ^c^Department of Pure Mathematics and Mathematical Statistics, Cambridge University, Cambridge CB3 0WA, United Kingdom

**Keywords:** number theory, modular forms, Galois representations

## Abstract

We study an analog of Serre’s modularity conjecture for projective representations $\bar{\rho }:\text{Gal}\left(\bar{K}/K\right)\to {PGL}_{2}\left(k\right)$, where K is a totally real number field. We prove cases of this conjecture when $k=F_{5}$.

## Significance

The connection between modular forms and Galois representations plays a significant role in modern algebraic number theory. J.-P. Serre made an influential conjecture relating mod p modular forms and mod p representations of the absolute Galois group of Q. Such a relationship has consequences for classical Diophantine questions, for example implying Fermat’s Last Theorem, and is also a mod p analogue of the Langlands program. It is thus important to study analogues of Serre’s conjecture in the broadest possible context. Serre’s modularity conjecture, definitively stated in 1986, was proved by Khare–Wintenberger in 2009. In this paper we prove new cases of extensions of Serre’s conjecture to mod p representations of absolute Galois groups of totally real number fields.

Let K be a number field, and consider a continuous representation

$$\rho :G_{K}\to {GL}_{2}\left(k\right),$$

where k is a finite field. (Here $G_{K}$ denotes the absolute Galois group of K; for this and other notation, see *1.A. Notation* below.) We say that ρ is of Serre type, or S type, if it is absolutely irreducible and totally odd, in the sense that for each real place v of K and each associated complex conjugation $c_{v}\in G_{K}$, $\det\rho \left(c_{v}\right)=-1$.

Serre’s conjecture and its generalizations assert that any ρ of S type should be automorphic (see for example refs. 1 and 2 in the case K=Q, ref. 3 when K is totally real, and ref. 4 for a general number field K). The meaning of the word “automorphic” depends on the context but when K is totally real, for example, we can ask for ρ to be associated to a cuspidal automorphic representation π of ${GL}_{2}\left(A_{K}\right)$, which is regular algebraic of weight 0 (*2.A. Automorphy of Linear and Projective Representations*). Serre’s conjecture is now a theorem when K=Q (5, 6). For a totally real field K, some results are available when k is “small.” These are summarized in *Theorem 1.1*, which relies upon refs. 2 and 7–10:

## Theorem 1.1.

*Let*K*be a totally real number field*, *and let*$\rho :G_{K}\to {GL}_{2}\left(k\right)$*be a representation of*S type. *Then* ρ *is automorphic provided* |k|∈{2,3,4,5,7,9}.

One can equally consider continuous representations

$$\sigma :G_{K}\to {PGL}_{2}\left(k\right),$$

where again k is a finite field. We say that σ is of S type if it is absolutely irreducible and totally odd, in the sense that if k has odd characteristic, then for each real place v of K, $\sigma \left(c_{v}\right)$ is nontrivial. One could formulate a projective analog of Serre’s conjecture, asking that any representation σ of S type be automorphic. A theorem of Tate implies that σ lifts to a linear representation valued in ${GL}_{2}\left(k^{\prime }\right)$ for some finite extension $k^{\prime }/k$, and by σ being automorphic we mean that a lift of it to a linear representation is automorphic (*2.A. Automorphy of Linear and Projective Representations*). Thus, if k is allowed to vary, this conjecture is equivalent to Serre’s conjecture, since any representation ρ has an associated projective representation Proj(ρ), and any projective representation σ lifts to a representation valued in ${GL}_{2}\left(k^{\prime }\right)$ for some finite extension $k^{\prime }/k$; moreover, ρ is of S type if and only if Proj(ρ) is, and ρ is automorphic if and only if Proj(ρ) is.

However, for fixed k the two conjectures are not equivalent: Certainly if ρ is valued in ${GL}_{2}\left(k\right)$ then Proj(ρ) takes values in ${PGL}_{2}\left(k\right)$, but it is not true that any representation $\sigma :G_{K}\to {PGL}_{2}\left(k\right)$ admits a lift valued in ${GL}_{2}\left(k\right)$, and in fact in general the determination of the minimal extension $k^{\prime }/k$ such that there is a lift to ${GL}_{2}\left(k^{\prime }\right)$ is somewhat subtle. It is therefore of interest to ask whether the consideration of projective representations allows one to expand the list of “known” cases of Serre’s conjecture.

Our main theorem affirms that this is indeed the case. Before giving the statement we need to introduce one more piece of notation. We write $\Delta :{PGL}_{2}\left(k\right)\to k^{\times }/{\left(k^{\times }\right)}^{2}$ for the homomorphism induced by the determinant. We say that a homomorphism $G_{K}\to k^{\times }/{\left(k^{\times }\right)}^{2}$ is totally even (resp. totally odd) if each complex conjugation in $G_{K}$ is a trivial (resp. nontrivial) image.

## Theorem 1.2.

*Let*K*be a totally real number field*, *and let*$\sigma :G_{K}\to {PGL}_{2}\left(k\right)$*be a representation of*S*type*. *Then*σ*is automorphic provided that one of the following conditions is satisfied*:

1) |k|∈{2,3,4}.
2) |k|=5, $\left[K\left(\zeta_{5}\right):K\right]=4$, *and*Δ○σ*is totally even.*
3) |k|=5, $\left[K\left(\zeta_{5}\right):K\right]=4$, *and*Δ○σ*is totally odd.*
4) |k|=7*and*Δ○σ*is totally odd.*
5) |k|=9*and*Δ○σ*is totally even.*

We note the exceptional isomorphisms ${PSL}_{2}\left(F_{9}\right)=A_{6}$, ${PGL}_{2}\left(F_{5}\right)=S_{5}$, ${PGL}_{2}\left(F_{3}\right)=S_{4}$, ${PGL}_{2}\left(F_{2}\right)=S_{3}$, which link our results to showing that splitting fields of polynomials of small degree over K arise automorphically.

The proof of *Theorem 1.2* falls into three cases. The first one is when |k| is even or $k=F_{3}$. When |k| is even, the homomorphism ${GL}_{2}\left(k\right)\to {PGL}_{2}\left(k\right)$ splits, so we reduce easily to *Theorem 1.1*. When $k=F_{3}$, the homomorphism ${PGL}_{2}\left(Z\left[\sqrt{-2}\right]\right)\to {PGL}_{2}\left(F_{3}\right)$ splits and we can use the Langlands–Tunnell theorem (7) to establish the automorphy of σ.

The second case is when |k| is odd and −1 is a square in k (resp. a nonsquare in k) and Δ○σ is totally even (resp. totally odd). In this case we are able to construct the following data:

• A solvable totally real extension L/K and a representation ${\bar{\rho }}_{1}:G_{L}\to {GL}_{2}\left(k\right)$ such that $Proj\left({\bar{\rho }}_{1}\right)=\sigma {|}_{G_{L}}$ (by showing that L/K can be chosen to kill the Galois cohomological obstruction to lifting).
• A representation $\rho_{2}:G_{K}\to {GL}_{2}\left({\bar{Q}}_{p}\right)$ such that $Proj\left({\bar{\rho }}_{2}\right)$ and σ are conjugate in ${PGL}_{2}\left({\bar{F}}_{p}\right)$ (by choosing an arbitrary lift of σ to ${GL}_{2}\left({\bar{F}}_{p}\right)$ and applying the Khare–Wintenberger method).

We can then use *Theorem 1.1* to verify the automorphy of ${\bar{\rho }}_{1}$ and hence the residual automorphy of ${\bar{\rho }}_{2}{|}_{G_{L}}$. An automorphy lifting theorem then implies the automorphy of $\rho_{2}{|}_{G_{L}}$, hence $\rho_{2}$ itself by solvable descent, and hence finally of σ.

The final case is when $k=F_{5}$ and Δ○σ is totally odd. In this case there does not exist any totally real extension L/K such that $\sigma {|}_{G_{L}}$ lifts to a representation valued in ${GL}_{2}\left(k\right)$ (there is a local obstruction at the real places). However, it is possible to find a CM extension L/K such that $\sigma {|}_{G_{L}}$ lifts to a representation valued in ${GL}_{2}\left(k\right)$ with determinant the cyclotomic character. (By definition, a CM number field is a quadratic, totally imaginary extension of a totally real field.) When $k=F_{5}$, such a representation necessarily appears in the group of 5-torsion points of an elliptic curve over L (8) and so we can use the automorphy results over CM fields established in ref. 11 together with a solvable descent argument to obtain the automorphy of σ.

### Remark 1.3:

In the final case above of a representation $\sigma :G_{K}\to {PGL}_{2}\left(F_{5}\right)$ with nonsolvable image, the residual automorphy of the lift $\rho :G_{L}\to {GL}_{2}\left(F_{5}\right)$ ultimately depends on ref. 11, theorem 7.1, which proves the automorphy of certain residually dihedral 2-adic Galois representations. The residual automorphy of these 2-adic representations is verified using automorphic induction. In particular, our proof in this case does not depend on the use of the Langlands–Tunnell theorem. This is in contrast to the argument used in, e.g., ref. 8, theorem 4.1 to establish the automorphy of representations $\rho^{\prime }:G_{K}\to {GL}_{2}\left(F_{5}\right)$ with cyclotomic determinant.

This “2–3 switch” strategy can also be used to prove the automorphy of representations $\sigma :G_{K}\to {PGL}_{2}\left(F_{3}\right)$ with Δ○σ totally odd using the 2-adic automorphy theorems proved in ref. 12; see *Theorem 3.1*. This class of representations includes the projective representations associated to the Galois action on the 3-torsion points of an elliptic curve over K. This gives a way to verify the modulo 3 residual automorphy of elliptic curves over K, which does not rely on the Langlands–Tunnell theorem (and in particular refs. 13 and 14) but only on the Saito–Shintani lifting for holomorphic Hilbert modular forms (15). (We note that we do need to use the Langlands–Tunnell theorem to prove the automorphy of representations $\sigma :G_{K}\to {PGL}_{2}\left(F_{3}\right)$ with Δ○σ totally even; cf. *Theorem 2.12*.)

We now describe the structure of this paper. We begin in *2. Lifting Representations* by studying the lifts of projective representations and collecting various results about the existence of characteristic 0 lifts of residual representations and their automorphy. We are then able to give the proofs of *Theorem 1.1* and the first two cases in the proof of *Theorem 1.2* described above. In *3. Modularity of Mod 3 Representations* we expand on *Remark 1.3* by showing how the main theorems of ref. 12 can be used to give another proof of the automorphy of S-type representations $\sigma :G_{K}\to {PGL}_{2}\left(F_{3}\right)$ (still under the hypothesis that K is totally real and Δ○σ is totally odd). Finally, in *4. Modularity of Mod 5 Representations* we use similar arguments, now based on the main theorems of ref. 11, to complete the proof of *Theorem 1.2*.

## A. Notation.

If K is a perfect field, then we write $G_{K}=\text{Gal}\left(\bar{K}/K\right)$ for the Galois group of K with respect to a fixed choice of algebraic closure. If K is a number field and v is a place of K, then we write $K_{v}$ for the completion of K at v and fix an embedding $\bar{K}\to {\bar{K}}_{v}$ extending the natural embedding $K\to K_{v}$; this determines an injective homomorphism $G_{K_{v}}\to G_{K}$. If v is a finite place of K, then we write ${\text{Frob}}_{v}\in G_{K_{v}}$ for a lift of the geometric Frobenius, k(v) for the residue field of $K_{v}$, and $q_{v}$ for the cardinality of $K_{v}$; if v is a real place, then we write $c_{v}\in G_{K_{v}}$ for complex conjugation. Any homomorphism from a Galois group $G_{K}$ to another topological group will be assumed to be continuous.

If p is a prime and K is a field of characteristic 0, then we write $\epsilon :G_{K}\to Z_{p}^{\times }$ for the p-adic cyclotomic character, $\bar{\epsilon }:G_{K}\to F_{p}^{\times }$ for its reduction modulo p, and $\omega :G_{K}\to F_{p}^{\times }/{\left(F_{p}^{\times }\right)}^{2}$ for the character $\bar{\epsilon }\text{mod}{\left(F_{p}^{\times }\right)}^{2}$. More generally, if $\rho :G_{K}\to {GL}_{n}\left({\bar{Q}}_{p}\right)$ is a representation, then we write $\bar{\rho }:G_{K}\to {GL}_{n}\left({\bar{F}}_{p}\right)$ for the associated semisimple residual representation (uniquely determined up to conjugation).

If k is a field, then we write $Proj:{GL}_{n}\left(k\right)\to {PGL}_{n}\left(k\right)$ for the natural projection and $\Delta :{PGL}_{n}\left(k\right)\to k^{\times }/{\left(k^{\times }\right)}^{n}$ for the character induced by the determinant. We use these maps only in the case n=2.

If K is a field of characteristic 0, E is an elliptic curve over K, and p is a prime, then we write ${\bar{\rho }}_{E,p}:G_{K}\to {GL}_{2}\left(F_{p}\right)$ for the representation associated to $H^{1}\left(E_{\bar{K}},F_{p}\right)$ after a choice of basis. Thus $\det{\bar{\rho }}_{E,p}={\bar{\epsilon }}^{-1}$.

## 2. Lifting Representations

In this section we study different kinds of liftings of representations: liftings to characteristic 0 (and the automorphy of such liftings) and liftings of projective representations to true (linear) representations. We begin by discussing what it means for a (projective or linear) representation to be automorphic.

### A. Automorphy of Linear and Projective Representations.

Let K be a CM or totally real number field. If π is a cuspidal, regular algebraic automorphic representation of ${GL}_{2}\left(A_{K}\right)$, then (16, 17) for any isomorphism $\iota :{\bar{Q}}_{p}\to C$, there exists a semisimple representation $r_{\iota }\left(\pi \right):G_{K}\to {GL}_{2}\left({\bar{Q}}_{p}\right)$ satisfying the following condition, which determines $r_{\iota }\left(\pi \right)$ uniquely up to conjugation: For all but finitely many finite places v of K such that $\pi_{v}$ is unramified, $r_{\iota }\left(\pi \right){|}_{G_{K_{v}}}$ is unramified and $r_{\iota }\left(\pi \right){|}_{G_{K_{v}}}^{ss}$ is related to the representation $\iota^{-1}\pi_{v}$ under the Tate-normalized unramified local Langlands correspondence. (See ref. 18, section 2 for an explanation of how the characteristic polynomial of $r_{\iota }\left(\pi \right){|}_{G_{K_{v}}}$ may be expressed in terms of the eigenvalues’ explicit unramified Hecke operators.) In this paper we need only to consider automorphic representations that are of regular algebraic automorphic representations π that are of weight 0, in the sense that for each place v|∞ of K, $\pi_{v}$ has the same infinitesimal character as the trivial representation.

Let k be a finite field of characteristic p, viewed inside its algebraic closure ${\bar{F}}_{p}$. In this paper, we say that a representation $\rho :G_{K}\to {GL}_{2}\left(k\right)$ is automorphic if it is ${GL}_{2}\left({\bar{F}}_{p}\right)$ conjugate to a representation of the form $\bar{r_{\iota }\left(\pi \right)}$, where π is a cuspidal, regular algebraic automorphic representation of ${GL}_{2}\left(A_{K}\right)$ of weight 0. We say that a representation $\sigma :G_{K}\to {PGL}_{2}\left(k\right)$ is automorphic if it is ${PGL}_{2}\left({\bar{F}}_{p}\right)$ conjugate to a representation of the form $Proj\left(\bar{r_{\iota }\left(\pi \right)}\right)$, where π is a cuspidal, regular algebraic automorphic representation of ${GL}_{2}\left(A_{K}\right)$ of weight 0.

We say that a representation $\rho :G_{K}\to {GL}_{2}\left({\bar{Q}}_{p}\right)$ is automorphic if it is conjugate to a representation of the form $\bar{r_{\iota }\left(\pi \right)}$, where π is a cuspidal, regular algebraic automorphic representation of ${GL}_{2}\left(A_{K}\right)$ of weight 0. We say that an elliptic curve E over K is modular if the representation of $G_{K}$ afforded by $H^{1}\left(E_{\bar{K}},Q_{p}\right)$ is automorphic in this sense.

### Lemma 2.1.

*Let*K*be a CM or totally real number field*, *let*$\rho :G_{K}\to {GL}_{2}\left(k\right)$*be a representation*, *and let*σ=Proj(ρ). *Then*,

1) *Let*$\chi :G_{K}\to k^{\times }$*be a character. Then*ρ*is automorphic if and only if*ρ⊗χ*is automorphic.*
2) σ*is automorphic if and only if*ρ*is automorphic.*

#### Proof:

If $\chi :G_{K}\to k^{\times }$ is a character, then its Teichmüller lift $X:G_{K}\to {\bar{Q}}_{p}^{\times }$ is associated, by class field theory, to a finite-order Hecke character $\Xi :A_{K}^{\times }\to C^{\times }$. If π is a cuspidal automorphic representation that is regular algebraic of weight 0 and $\bar{r_{\iota }\left(\pi \right)}$ is conjugate to ρ, then π⊗(Ξ○det) is also cuspidal and regular algebraic of weight 0 and $\bar{r_{\iota }\left(\pi ⊗\left(\Xi ○\det\right)\right)}$ is conjugate to ρ⊗χ.

It is clear from the definition that if ρ is automorphic, then so is σ. Conversely, if σ is automorphic, then there is a cuspidal, regular algebraic automorphic representation π of ${GL}_{2}\left(A_{K}\right)$ and isomorphism $\iota :{\bar{Q}}_{p}\to C$ such that $Proj\left(\bar{r_{\iota }\left(\pi \right)}\right)=Proj\left(\rho \right)$. It follows that there exists a character $\chi :G_{K}\to {\bar{F}}_{p}^{\times }$ such that ρ is conjugate to $\bar{r_{\iota }\left(\pi \right)}⊗\chi$. The automorphy of ρ follows from the first part of *Lemma 2.1*.

### B. Lifting to Characteristic 0.

We recall a result on the existence of liftings with prescribed properties. We first need to say what it means for a representation to be exceptional. If K is a number field and $\sigma :G_{K}\to {PGL}_{2}\left(k\right)$ is a projective representation, we say that σ is exceptional if it is ${PGL}_{2}\left({\bar{F}}_{p}\right)$ conjugate to a representation $\sigma^{\prime }:G_{K}\to {PGL}_{2}\left(F_{5}\right)$ such that $\sigma^{\prime }\left(G_{K}\right)$ contains ${PSL}_{2}\left(F_{5}\right)$ and the character ${\left(-1\right)}^{\Delta ○\sigma^{\prime }}\bar{\epsilon }$ is trivial. [Here we write ${\left(-1\right)}^{\Delta ○\sigma^{\prime }}$ for the composition of $\Delta ○\sigma^{\prime }$ with the unique isomorphism $F_{5}^{\times }/{\left(F_{5}^{\times }\right)}^{2}≅\left\{\pm 1\right\}$.] We say that a representation $\rho :G_{K}\to {GL}_{2}\left(k\right)$ is exceptional if Proj(ρ) is exceptional. If K is totally real, then this is equivalent to the definition given in ref. 19, section 3. The exceptional case is often excluded in the statements of automorphy lifting theorems (the root cause being the nontriviality of the group $H^{1}\left(\sigma \left(G_{K}\right),{\text{Ad}}^{0}\rho \left(1\right)\right)$).

### Theorem 2.2.

*Let*K*be a totally real field*, *let*$\bar{\rho }:G_{K}\to {GL}_{2}\left(k\right)$*be a representation of*S*type*, *and let*$\psi :G_{K}\to {\bar{Z}}_{p}^{\times }$*be a continuous character lifting*$\det\bar{\rho }$*such that*ψϵ*is of finite order*. *Suppose that the following conditions are satisfied*:

1) p>2*and*$\bar{\rho }{|}_{G_{K\left(\zeta_{p}\right)}}$*is absolutely irreducible.*
2) *If*p=5, *then*$\bar{\rho }$*is nonexceptional.*

*Then*$\bar{\rho }$*lifts to a continuous representation*$\rho :G_{K}\to {GL}_{2}\left({\bar{Z}}_{p}\right)$*satisfying the following conditions*:

1) *For all but finitely many places*v*of*F, $\rho {|}_{G_{K_{v}}}$*is unramified.*
2) detρ=ψ.
3) *For each place*v|p*of*K, $\rho {|}_{G_{K_{v}}}$*is potentially crystalline and for each embedding*$\tau :K_{v}\to {\bar{Q}}_{p}$, ${HT}_{\tau }\left(\rho \right)=\left\{0,1\right\}$. *Moreover*, *for any*v|p*such that*$\bar{\rho }{|}_{G_{K_{v}}}$*is reducible*, *we can assume that*$\rho {|}_{G_{K_{v}}}$*is ordinary*, *in the sense of ref.*^18^, *section* 5.1.

#### Proof:

This follows from ref. 20, theorem 7.6.1, on noting that the condition (A2) there can be replaced by the more general condition that $\bar{\rho }$ is nonexceptional [indeed, the condition (A2) is used to invoke ref. 21, proposition 3.2.5, which is proved under this more general condition]. To verify the existence of a potentially crystalline lift of $\bar{\rho }{|}_{G_{K_{v}}}$ for each v|p (or in the terminology of loc. cit., the compatibility of $\bar{\rho }{|}_{G_{K_{v}}}$ with type A or B) we apply ref. 20, proposition 7.8.1 (when $\bar{\rho }{|}_{G_{K_{v}}}$ is irreducible) or ref. 22, lemma 6.1.6 (when $\bar{\rho }{|}_{G_{K_{v}}}$ is reducible).

We next recall an automorphy lifting theorem.

### Theorem 2.3.

*Let*K*be a totally real number field*, *and let*$\rho :G_{K}\to {GL}_{2}\left({\bar{Z}}_{p}\right)$*be a continuous representation satisfying the following conditions*:

1) p>2*and*$\bar{\rho }{|}_{G_{K\left(\zeta_{p}\right)}}$*is absolutely irreducible.*
2) *For all but finitely many finite places*v*of*K, $\rho {|}_{G_{K_{v}}}$*is unramified.*
3) *For each place*v|p*of*K, $\rho {|}_{G_{K_{v}}}$*is de Rham and for each embedding*$\tau :K_{v}\to {\bar{Q}}_{p}$, ${HT}_{\tau }\left(\rho \right)=\left\{0,1\right\}$.
4) *The representation*$\bar{\rho }$*is automorphic.*

*Then*ρ*is automorphic*.

#### Proof:

This follows from ref. 19, theorem 9.3.

We now combine the previous two theorems to obtain a “solvable descent of automorphy” theorem for residual representations, along similar lines to refs. 23 and 24.

### Proposition 2.4.

*Let*K*be a totally real number field and let*$\bar{\rho }:G_{K}\to {GL}_{2}\left(k\right)$*be a representation of*S*type*. *Suppose that there exists a solvable totally real extension*L/K*such that the following conditions are satisfied*:

1) p>2*and*$\bar{\rho }{|}_{G_{L\left(\zeta_{p}\right)}}$*is absolutely irreducible. If*p=5, *then*$\bar{\rho }$*is nonexceptional.*
2) $\bar{\rho }{|}_{G_{L}}$*is automorphic.*

*Then*$\bar{\rho }$*is automorphic*.

#### Proof:

Let $\psi :G_{K}\to {\bar{Z}}_{p}^{\times }$ be the character such that ψϵ is the Teichmüller lift of $\left(\det\bar{\rho }\right)\bar{\epsilon }$, and let $\rho :G_{K}\to {GL}_{2}\left({\bar{Z}}_{p}\right)$ be the lift of ρ whose existence is asserted by *Theorem 2.2*. Then *Theorem 2.3* implies the automorphy of $\rho {|}_{G_{L}}$, and the automorphy of ρ itself and hence of $\bar{\rho }$ follows by cyclic descent, using the results of Langlands (13).

We can now give the proof of *Theorem 1.1*, which we restate here for the convenience of the reader:

### Theorem 2.5.

*Let*K*be a totally real field and let*$\bar{\rho }:G_{K}\to {GL}_{2}\left(k\right)$*be a representation of*S*type*. *Suppose that*|k|∈{2,3,4,5,7,9}. *Then*$\bar{\rho }$*is automorphic*.

#### Proof:

Many of the results we quote here are stated in the case of K=Q but hold more generally for totally real fields with minor modification. We apply them in the more general setting without further comment.

If $\bar{\rho }$ is dihedral, then this is a consequence of results of Hecke (ref. 2, section 5.1). If $k=F_{3}$, it is a consequence of the Langlands–Tunnell theorem (7) (see the discussion following theorem 5.1 in ref. 25, chap. 5). We may thus assume for the remainder of the proof that |k|>3. We may also assume that for any abelian extension L/K, the restriction $\bar{\rho }{|}_{G_{L\left(\zeta_{p}\right)}}$ is absolutely irreducible (as otherwise $\bar{\rho }$ would be dihedral).

Next suppose that $k=F_{5}$. We note that $\bar{\rho }$ is not exceptional, by ref. 19, lemma 3.1. Let L/K be the totally real cyclic extension cut out by $\left(\det\bar{\rho }\right)\bar{\epsilon }$. By ref. 8, theorem 1.2, there is an elliptic curve E over L such that ${\bar{\rho }}_{E,5}≅\bar{\rho }{|}_{G_{L}}$ and ${\bar{\rho }}_{E,3}\left(G_{L}\right)$ contains ${SL}_{2}\left(F_{3}\right)$. By the $k=F_{3}$ case of the theorem and by *Theorem 2.3*, we see that E is automorphic and hence so is $\bar{\rho }{|}_{G_{L}}$. The automorphy of $\bar{\rho }$ then follows from *Proposition 2.4*. The $k=F_{7}$ case is similar, using ref. 9, proposition 3.1 instead of ref. 8, theorem 1.2.

Next suppose that $k=F_{4}$. We can twist ρ to assume that it is valued in ${SL}_{2}\left(F_{4}\right)$. Then ref. 8, theorem 3.4 shows that there is an abelian surface A over F with real multiplication by $O_{Q\left(\sqrt{5}\right)}$ such that the $G_{K}$ representation on $A\left[2\right]≅F_{4}^{2}$ is isomorphic to $\bar{\rho }$ and such that the $G_{K}$ representation on $A\left[\sqrt{5}\right]≅F_{5}^{2}$ has an image containing ${SL}_{2}\left(F_{5}\right)$. By the $k=F_{5}$ case of the theorem, *Theorems 2.2* and *2.3*, we see that A is automorphic, and hence so is $\bar{\rho }$.

Finally suppose that $k=F_{9}$. Let L/K be the totally real cyclic extension cut out by $\left(\det\bar{\rho }\right)\bar{\epsilon }$. Then the argument of ref. 10, section 2.5 shows that there is a solvable totally real extension M/K containing L/K and an abelian surface A over M with real multiplication by $O_{Q\left(\sqrt{5}\right)}$ such that the $G_{M}$ representation on $A\left[3\right]≅F_{9}^{2}$ is isomorphic to $\bar{\rho }{|}_{G_{M}}⊗\bar{\epsilon }$ and such that the $G_{M}$ representation on $A\left[\sqrt{5}\right]≅F_{5}^{2}$ has an image containing ${SL}_{2}\left(F_{5}\right)$. By the $k=F_{5}$ case of the theorem, *Theorems 2.2* and *2.3*, we see that A is automorphic, and hence so is $\bar{\rho }{|}_{G_{M}}$. The automorphy of $\bar{\rho }$ follows from *Proposition 2.4*.

#### Remark 2.6:

The 2–3 switch strategy employed in *Theorem 3.1* below can be used to prove automorphy of totally odd representations $\rho :G_{K}\to {GL}_{2}\left(F_{3}\right)$ without using the Langlands–Tunnell theorem.

### C. Lifting Projective Representations.

We now consider the problem of lifting projective representations.

### Lemma 2.7.

*Let*K*be a number field*, *and let*$\sigma :G_{K}\to {PGL}_{2}\left(k\right)$*be a continuous homomorphism*. *Then there exists a finite extension*$k^{\prime }/k$*such that*σ*lifts to a homomorphism*$\rho :G_{K}\to {GL}_{2}\left(k^{\prime }\right)$.

#### Proof:

The obstruction to lifting a continuous homomorphism $\sigma :G_{K}\to {PGL}_{2}\left(k\right)$ to a continuous homomorphism $\rho :G_{K}\to {GL}_{2}\left(\bar{k}\right)$ lies in $H^{2}\left(G_{K},{\bar{k}}^{\times }\right)$. Tate proved that $H^{2}\left(G_{K},{\bar{k}}^{\times }\right)=0$ (ref. 26, section 6.5), so a lift always exists.

### Lemma 2.8.

*Suppose that*p>2, *let*K*be a number field*, *and let*$\sigma :G_{K}\to {PGL}_{2}\left(k\right)$*be a homomorphism*. *Let*S*be a finite set of places of*K*such that for each*v∈S, *there exists a lift of*$\sigma {|}_{G_{K_{v}}}$*to a homomorphism*$\rho_{v}:G_{K}\to {GL}_{2}\left(k\right)$. *Then we can find the following data*:

1) *A solvable*S-*split extension*L/K.
2) *A homomorphism*$\rho :G_{L}\to {GL}_{2}\left(k\right)$*such that*$Proj\left(\rho \right)=\sigma {|}_{G_{L}}$*and for each*v∈S*and each place*w|v*of*L, $\rho {|}_{G_{L_{w}}}=\rho_{v}$.

*Moreover*, *if*K*is a CM field*, *we can choose*L*also to be a CM field*.

#### Proof:

Let H denote the 2-Sylow subgroup of $k^{\times }$, of order $2^{m}$, and let $H^{\prime }\leq k^{\times }$ denote its prime-to-2 complement. If 0≤k≤m, we write $G_{k}={GL}_{2}\left(k\right)/\left(2^{m-k}H\times H^{\prime }\right)$, which is an extension

$$1\to H/2^{m-k}H\to G_{k}\to {PGL}_{2}\left(k\right)\to 1.$$

We show by induction on k≥0 that we can find a solvable, S-split extension $L_{k}/K$ and a homomorphism $\rho_{k}:G_{L_{k}}\to G_{k}$ lifting $\sigma {|}_{G_{L_{k}}}$ and such that for each v∈S and each place w|v of $L_{k}$, $\rho_{k}{|}_{G_{L_{k,w}}}=\rho_{v}\text{mod}2^{m-k}H\times H^{\prime }$. The case k=0 is the existence of σ. The case k=m implies the statement of the lemma, since ${GL}_{2}\left(k\right)=G_{m}\times H^{\prime }$. (Note that in ref. 27, chap. X, theorem 5 implies that any collection of characters $\chi_{v}:G_{K_{v}}\to H^{\prime }$ can be globalized to a character $\chi :G_{K}\to H^{\prime }$.)

For the induction step, suppose the induction hypothesis holds for a fixed value of k. We consider the obstruction to lifting $\rho_{k}$ to a homomorphism $\rho_{k+1}:G_{L_{k}}\to G_{k+1}$. This defines an element of $H^{2}\left(G_{L_{k}},Z/2Z\right)$, which is locally trivial at the places of $L_{k}$ lying above S. We can therefore find an extension of the form $L_{k+1}=L_{k}\cdot E_{k+1}$, where $E_{k+1}/K$ is a solvable S-split extension, such that the image of this obstruction class in $H^{2}\left(G_{L_{k+1}},Z/2Z\right)$ vanishes and so there is a homomorphism $\rho_{k+1}^{\prime }:G_{L_{k+1}}\to G_{k+1}$ lifting $\rho_{k}{|}_{G_{L_{k+1}}}$.

If v∈S and w|v is a place of $L_{k+1}$, then there is a character $\chi_{w}:G_{L_{k+1,w}}\to Z/2Z$ such that $\rho_{k+1}^{\prime }{|}_{G_{L_{k+1,w}}}=\left(\rho_{v}\text{mod}2^{m-\left(k+1\right)}H\times H^{\prime }\right)\cdot \chi_{w}$. We can certainly find a character $\chi :G_{L_{k+1}}\to Z/2Z$ such that $\chi {|}_{G_{L_{k+1,w}}}=\chi_{w}$ for each such place w. The induction step is complete on taking $\rho_{k+1}=\rho_{k+1}^{\prime }\cdot \chi$.

It remains to explain why we can choose K to be CM if L is. Since the extensions $E_{k}$ in the proof are required only to satisfy some local conditions, which are vacuous if K is CM, we can choose the fields $E_{k}$ to be of the form $KE_{k}^{\prime }$, where $E_{k}^{\prime }$ is a totally real extension, in which case the field L constructed in the proof is seen to be CM.

#### Remark 2.9:

We remark that if v is a real place of K and $\sigma \left(c_{v}\right)\neq 1$, then there exists a lift of $\sigma {|}_{G_{K_{v}}}$ to ${GL}_{2}\left(k\right)$ if and only if either −1 is a square in $k^{\times }$ and $\Delta ○\sigma \left(c_{v}\right)=1$ or −1 is not a square in $k^{\times }$ and $\Delta ○\sigma \left(c_{v}\right)\neq 1$. We also note the utility of the “S-split” condition: We can add any set of places at which σ is unramified to S and in this way ensure that the S-split extension L/K is linearly disjoint from any other fixed finite extension of K.

Here is a variant.

### Lemma 2.10.

*Suppose that*p>2. *Let*K*be a number field*, *let*$\sigma :G_{K}\to {PGL}_{2}\left(k\right)$*be a homomorphism*, *and let*$\chi :G_{K}\to k^{\times }$*be a character*. *Suppose that the following conditions are satisfied*:

1) $\Delta ○\sigma =\chi \text{mod}{\left(k^{\times }\right)}^{2}$.
2) *For each finite place*v*of*K, $\sigma {|}_{G_{K_{v}}}$*and*$\chi {|}_{G_{K_{v}}}$*are unramified.*
3) *For each real place*v*of*K, $\sigma \left(c_{v}\right)\neq 1$*and*$\chi \left(c_{v}\right)=-1$.

*Then there exists a homomorphism*$\rho :G_{K}\to {GL}_{2}\left(k\right)$*such that*Proj(ρ)=σ*and*det(ρ)=χ.

#### Proof:

We consider the short exact sequence of groups

$$1\to \left\{\pm 1\right\}\to {GL}_{2}\left(k\right)\to {PGL}_{2}\left(k\right)\times_{\Delta }k^{\times }\to 1,$$

where the last group is the subgroup of $\left(g,\alpha \right)\in {PGL}_{2}\left(k\right)\times k^{\times }$ such that $\Delta \left(g\right)=\alpha \text{mod}{\left(k^{\times }\right)}^{2}$. By hypothesis the pair (σ,χ) defines a homomorphism $\Sigma :G_{K}\to {PGL}_{2}\left(k\right)\times_{\Delta }k^{\times }$ such that for every place v of K, $\Sigma {|}_{G_{K_{v}}}$ lifts to ${GL}_{2}\left(k\right)$ (*Remark 2.9*). The subgroup of locally trivial elements of $H^{2}\left(G_{K},\left\{\pm 1\right\}\right)$ is trivial, by class field theory, so Σ lifts to a homomorphism $\rho :G_{K}\to {GL}_{2}\left(k\right)$, as required.

We now prove an analog of *Proposition 2.4* for projective representations.

### Proposition 2.11.

*Let*K*be a totally real number field and let*$\sigma :G_{K}\to {PGL}_{2}\left(k\right)$*be a representation of*S*type*. *Suppose that there exists a solvable totally real extension*L/K*satisfying the following conditions*:

1) p>2*and*$\sigma {|}_{G_{L\left(\zeta_{p}\right)}}$*is absolutely irreducible. If*p=5, *then*σ*is nonexceptional.*
2) $\sigma {|}_{G_{L}}$*is automorphic.*

*Then*σ*is automorphic*.

#### Proof:

By *Lemma 2.7*, we can lift σ to a representation $\bar{\rho }:G_{K}\to {GL}_{2}\left(\bar{k}\right)$. Then $\bar{\rho }{|}_{G_{L}}$ is automorphic and we can apply *Proposition 2.4* to conclude that $\bar{\rho }$ is automorphic and hence that σ is automorphic.

We are now in a position to establish a large part of *Theorem 1.2*.

### Theorem 2.12.

*Let*K*be a totally real number field and let*$\sigma :G_{K}\to {PGL}_{2}\left(k\right)$*be a representation of*S*type*. *If one of the following conditions holds*, *then*σ*is automorphic*:

1) |k|∈{2,3,4}.
2) |k|=5*or* 9 *and* Δ○σ *is totally even. If* |k|=5, *then* σ *is nonexceptional.*
3) |k|=7*and*Δ○σ*is totally odd.*

#### Proof:

When $k=F_{2}$ or $F_{4}$, the map ${SL}_{2}\left(k\right)\to {PGL}_{2}\left(k\right)$ is an isomorphism, so σ trivially lifts to a ${GL}_{2}\left(k\right)$ representation and we can apply *Theorem 2.5*. The case when |k|=3 follows from ref. 7. In the other cases, we can assume that $\sigma {|}_{G_{K\left(\zeta_{p}\right)}}$ is absolutely irreducible (as otherwise σ lifts to a dihedral representation). Let $S_{\infty }$ be the set of infinite places of K and choose a finite set $S^{\prime }$ of finite places of K at which σ is unramified such that $\text{Gal}\left({\bar{K}}^{\ker\left(\sigma {|}_{G_{K\left(\zeta_{p}\right)}}\right)}/K\right)$ is generated by ${\left\{{\text{Frob}}_{v}\right\}}_{v\in S^{\prime }}$. We can apply *Lemma 2.8*, see also *Remark 2.9*, with $S=S_{\infty }\cup S^{\prime }$ to find a solvable, totally real extension L/K such that σ lifts to a representation $\bar{\rho }:G_{L}\to {GL}_{2}\left(k\right)$ such that $\bar{\rho }{|}_{G_{L\left(\zeta_{p}\right)}}$ is absolutely irreducible and $\bar{\rho }$ is not exceptional if p=5. Then *Theorem 2.5* implies the automorphy of $\bar{\rho }$ and *Proposition 2.11* implies the automorphy of σ, as desired.

## 3. Modularity of Mod 3 Representations

In this section, which is a warmup for the next one, we give a proof of the following theorem that does not depend on the Langlands–Tunnell theorem:

### Theorem 3.1.

*Let*K*be a totally real number field*, *and let*$\sigma :G_{K}\to {PGL}_{2}\left(F_{3}\right)$*be a representation of*S*type such that*Δ○σ*is totally odd*. *Then*σ*is automorphic*.

#### Proof:

We can assume that σ is not dihedral; by the classification of finite subgroups of ${PGL}_{2}\left(F_{3}\right)$, we can therefore assume that $\sigma \left(G_{K}\right)$ contains ${PSL}_{2}\left(F_{3}\right)$. By *Proposition 2.11*, we can moreover assume, after replacing K by a solvable totally real extension, that σ is everywhere unramified and that for each place v|2 of K, $q_{v}\equiv 1\text{mod}3$ and $\sigma {|}_{G_{K_{v}}}$ is trivial.

### Lemma 3.2.

*There exists a solvable totally real extension*L/K*and a modular elliptic curve*E*over*L*satisfying the following conditions*:

1) $\sigma \left(G_{L}\right)$*contains*${PSL}_{2}\left(F_{3}\right)$. *In particular*, $\sigma {|}_{G_{L}}$*is of*S*type.*
2) *The homomorphism*$Proj\left({\bar{\rho }}_{E,3}\right)$*is*${PGL}_{2}\left({\bar{F}}_{3}\right)$*conjugate to*$\sigma {|}_{G_{L}}$.

#### Proof:

The character $\left(\Delta ○\sigma \right)\omega :G_{K}\to F_{3}^{\times }$ is totally even and so cuts out a totally real (trivial or quadratic) extension L/K, and $\sigma \left(G_{L}\right)$ contains ${PSL}_{2}\left(F_{3}\right)$ and satisfies $\Delta ○\sigma {|}_{G_{L}}=\omega$. Using *Lemma 2.10*, we can find a lift $\bar{\rho }:G_{L}\to {GL}_{2}\left(F_{3}\right)$ of $\sigma {|}_{G_{L}}$ satisfying the following conditions:

• $\det\bar{\rho }={\bar{\epsilon }}^{-1}$.
• For each place v|2 of L, $\bar{\rho }{|}_{G_{L}}$ is trivial.
• $\bar{\rho }\left(G_{L}\right)$ contains ${SL}_{2}\left(F_{3}\right)$. In particular, $\bar{\rho }{|}_{G_{L\left(\zeta_{3}\right)}}$ is absolutely irreducible.

We can then apply ref. 11, lemma 9.7 to conclude that there exists an elliptic curve E/L satisfying the following conditions:

• There is an isomorphism ${\bar{\rho }}_{E,3}≅\bar{\rho }$.
• For each place v|2 of L, E has multiplicative reduction at v and the valuation at v of the minimal discriminant of E is 3.
• ${\bar{\rho }}_{E,2}\left(G_{L}\right)={SL}_{2}\left(F_{2}\right)$.

Then ref. 12, p. 1237, corollary implies that E is modular, proving the lemma.

We see that $\sigma {|}_{G_{L}}$ is automorphic. We can then apply *Proposition 2.11* to conclude that σ itself is automorphic, as required.

## 4. Modularity of Mod 5 Representations

In this section we complete the proof of *Theorem 1.2* by proving *Theorem 4.1* below.

### Theorem 4.1.

*Let*K*be a totally real field*, *and let*$\sigma :G_{K}\to {PGL}_{2}\left(F_{5}\right)$*be a representation of*S*type that is nonexceptional and such that*Δ○σ*is totally odd*. *Then*σ*is automorphic*.

#### Proof:

By the classification of subgroups of ${PGL}_{2}\left(F_{5}\right)$, automorphic induction, and the Langlands–Tunnell theorem, we can assume that $\sigma \left(G_{K}\right)$ contains ${PSL}_{2}\left(F_{5}\right)$. By *Theorem 2.2*, *Lemma 2.7*, and *Proposition 2.11* we can assume, after possibly replacing K by a solvable totally real extension, that the following conditions are satisfied:

• There exists a representation $\bar{\rho }:G_{K}\to {GL}_{2}\left({\bar{F}}_{5}\right)$ such that $Proj\left(\bar{\rho }\right)$ is ${PGL}_{2}\left({\bar{F}}_{5}\right)$ conjugate to σ. Moreover, $\bar{\rho }$ is everywhere unramified.
• There exists a representation $\rho :G_{K}\to {GL}_{2}\left({\bar{Q}}_{5}\right)$ lifting $\bar{\rho }$, which is unramified almost everywhere.
• For each place v|5 of K, $\zeta_{5}\in K_{v}$, $\bar{\rho }{|}_{G_{K_{v}}}$ is trivial, and $\rho {|}_{G_{K_{v}}}$ is ordinary, in the sense of ref. 18, section 5.1.
• Let χ=detρ. Then χϵ has finite-order prime to 5 and for each finite place v of K, $\chi \epsilon {|}_{G_{K_{v}}}$ is unramified. In particular, $\bar{\chi }$ is everywhere unramified.

Let $K^{\prime }/K$ denote the quadratic CM extension cut out by the character (Δ○σ)ω.

### Lemma 4.2.

*The representation*$\bar{\rho }{|}_{G_{K^{\prime }}}$*is decomposed generic in the sense of ref*. 28, *definition* 4.3.1.

#### Proof:

It is enough to find a prime number l such that l splits in $K^{\prime }$ and for each place v|l of $K^{\prime }$, $q_{v}\equiv 1\text{mod}5$ and the eigenvalues of $\bar{\rho }\left({\text{Frob}}_{v}\right)$ are distinct. The argument of ref. 28, lemma 7.1.5 (3) will imply the existence of such a prime l if we can show that if $M=K^{\prime }\left(\zeta_{5}\right)$ and $\tilde{M}/Q$ is the Galois closure of M/Q, then $\sigma \left(G_{\tilde{M}}\right)$ contains ${PSL}_{2}\left(F_{5}\right)$. To see this, first let $\tilde{K}/Q$ be the Galois closure of K/Q. Then $\tilde{K}$ is totally real, and so $\sigma \left(G_{\tilde{K}}\right)=\sigma \left(G_{K}\right)={PGL}_{2}\left(F_{5}\right)$ because Δ○σ is totally odd. The extension $M\tilde{K}/\tilde{K}$ is abelian, so $\tilde{M}/\tilde{K}$ is abelian and $\sigma \left(G_{\tilde{M}}\right)$ must contain ${PSL}_{2}\left(F_{5}\right)$.

By construction, $\Delta ○\sigma {|}_{G_{K^{\prime }}}={\bar{\epsilon }}^{-1}\text{mod}{\left(F_{5}^{\times }\right)}^{2}$, so *by Lemma 2.10*, $\sigma {|}_{G_{K^{\prime }}}$ lifts to a continuous homomorphism $\tau :G_{K^{\prime }}\to {GL}_{2}\left(F_{5}\right)$ such that $\det\tau ={\bar{\epsilon }}^{-1}$. In particular, there is a character $\bar{\psi }:G_{K^{\prime }}\to {\bar{F}}_{5}^{\times }$ such that $\tau =\bar{\rho }{|}_{G_{K^{\prime }}}⊗\bar{\psi }$. Let ψ denote the Teichmüller lift of $\bar{\psi }$; then the determinant of $\rho {|}_{G_{K^{\prime }}}⊗\psi$ equals $\epsilon^{-1}$.

### Lemma 4.3.

*The representation*τ*satisfies the following conditions*:

1) $\tau {|}_{G_{K^{\prime }\left(\zeta_{5}\right)}}$*is absolutely irreducible and*τ*is nonexceptional.*
2) τ*is decomposed generic.*

#### Proof:

The representation $\tau {|}_{G_{K^{\prime }\left(\zeta_{5}\right)}}$ is absolutely irreducible because its projective image contains ${PSL}_{2}\left(F_{5}\right)$. If $\zeta_{5}\in K^{\prime }$, then $\sqrt{5}\in K$ and so $K^{\prime }=K\left(\Delta ○\sigma \right)=K\left(\zeta_{5}\right)$; this possibility is ruled out because σ is nonexceptional. It follows that τ is nonexceptional. The representation τ is decomposed generic because $\bar{\rho }{|}_{G_{K^{\prime }}}$ is (and this condition depends only on the associated projective representation).

Due to *Lemma 4.3*, we can apply ref. 11, lemma 9.7 and ref. 11, corollary 9.13 to conclude the existence of a modular elliptic curve E over $K^{\prime }$ such that ${\bar{\rho }}_{E,5}≅\tau$ and for each place v|5 of $K^{\prime }$, E has multiplicative reduction at the place v. We can then apply the automorphy lifting theorem (ref. 11, theorem 8.1) to conclude that $\rho {|}_{G_{K^{\prime }}}⊗\psi$ is automorphic and hence that $\rho {|}_{G_{K^{\prime }}}$ is automorphic. It follows by cyclic descent (13) that ρ and hence σ are also automorphic, and this completes the proof.

## Data Availability

There are no data underlying this work.
